# Adaptive Strategy to Change Firing Phases of Collided Nodes in Extended-Desync TDMA-Based MANETs

**DOI:** 10.3390/s21206776

**Published:** 2021-10-12

**Authors:** Cheol-Woong Lee, Gyu-Min Lee, Byeong-Hee Roh

**Affiliations:** 1 Department of AI Convergence Network, Ajou University, Suwon 16499, Korea; cjfdnd369@ajou.ac.kr; 2 Department of Computer Engineering, Ajou University, Suwon 16499, Korea; mybrand@ajou.ac.kr

**Keywords:** bio-inspired, Extended-Desync TDMA, Medium Access Control (MAC), Wireless Sensor Network (WSN), Mobile Ad-hoc Network (MANET)

## Abstract

As a multi-hop extension of the desynchronization-based TDMA (Desync-TDMA), the extended Desync-TDMA (Ext-Desync) with self-adapting property is proposed to overcome the limitations of existing CSMA/CA and dynamic TDMA-based schemes for Mobile Ad-hoc Networks (MANETs). However, existing studies overlooked the potential problem of firing message collisions caused by node movements, leading to the severe degradation of MANET networking performance. In this paper, we derive a mathematical model to evaluate the problem due to collisions of firing messages for moving nodes. With the derived model, we propose a method for a collided node to determine whether it changes its firing phase or not, adaptively in a distributed manner, by considering both the collision situation and the slot utilization. The comparative analysis between the proposed method and existing representative ones is also presented for various networking features. The performances of the proposed method are compared with CSMA/CA as well as other existing Ext-Desync-based schemes. The numerical results show that the proposed method achieved much faster resolution and higher slot utilization in collision situations than other Ext-Desync-based schemes. In addition, we also show that the proposed method outperformed the comparable methods, including CSMA/CA, in terms of packet delivery ratios and end-to-end delays.

## 1. Introduction

Mobile Ad-hoc Networks (MANETs) have features to enable dynamic network configuration and to support data delivery between mobile nodes without infrastructure, as in tactical or disaster environments [1,2]. Since multiple mobile nodes share channels, Medium Access Control (MAC) protocols play key roles in managing and operating MANETs. Most studies have adopted the Carrier Sense Multiple Access (CSMA) or the Time Division Multiple Access (TDMA)-based schemes as MAC protocols for MANETs [3].

Schemes based on CSMA employed an RTS/CTS mechanism to solve the hidden node problem in MANETs, which causes high overhead and makes it difficult to provide stable performance in a congested environment with a large amount of traffic [4]. For TDMA-based MAC protocols, which have been widely studied for MANET environments [5], the synchronization of the slot time among nodes is required, but it is still known as one of the most challenging tasks to be solved in the networking environments where nodes are moving [6]. The use of Non-Orthogonal Multiple Access (NOMA) for MANETs was proposed in [7]; however, it requires Base Stations (BSs) to relay communications among nodes.

As an alternative for wireless MAC protocols, biologically inspired (bio-inspired) approaches, which model the collective behavior of various species’ ecosystems, have been studied [8,9]. The desynchronization-based TDMA (Desync-TDMA) [10,11], inspired by the firefly’s habits, has been proposed as one of the bio-inspired MAC protocols for full-mesh Wireless Sensor Networks (WSNs).

Desync-TDMA does not require any central agent; instead, each node in a Desync-TDMA network sends firing messages periodically and allocates its slot in a distributed manner considering its and other nodes’ firing times. Desync-TDMA has been widely adopted and modified in many studies to enhance decentralized reservation-based scheduling and resource management schemes [12,13,14,15,16,17,18,19,20,21,22,23,24]. Since the studies related to Desync-TDMA have focused on single-hop wireless network environments with static nodes, they are not suitable to apply directly to multi-hop network environments.

To support multi-hop delivery of information, extended Desync-TDMA (Ext-Desync) [25] has been proposed by extending Desync-TDMA. Each node broadcasts firing messages including its one-hop neighbor list and their firing times. By referring to firing messages from other nodes, all nodes can know their two-hop neighbors and their relative firing times, which can solve the hidden terminal problem in multi-hop wireless networking. There have been also numerous studies to enhance multi-hop wireless networking performances based on Desync-TDMA or Ext-Desync [26,27,28,29,30,31,32,33,34].

However, since nodes are moving in MANET, collisions among firing messages may occur due to the hidden terminal problem’s occurrence by the moving nodes. In [25], nodes that detect collisions change their firing times with a certain fixed probability to solve the problem. As the mobility of nodes increases, the possibility of collisions increases, and nodes may change their firing times frequently. The frequent change of firing times due to such collisions may cause significant degradations of Ext-Desync networking performances [35].

As we will explain in detail in Section 3.1, the collision of firing message affects the degradation of slot utilization performances, resulting in the degradation of the packet delivery performances. Existing studies overlooked this problem in depth in their schemes, although it may seriously impact network performances.

This paper proposes an effective method for a collided node to determine whether to change its firing phase or not in order to resolve the collision situation optimally. The determination is carried out by considering both the collision situation and the slot utilization. The main contributions of this paper are as follows:

- We deal with the potential and critical problem that Ext-Desync-based schemes have when operated in MANETs, which has been overlooked in other studies. The problem definition in detail and its effect on the networking performances are illustrated in Section 3.1.
- We derive an analytical model to evaluate the problem mathematically. Then, we also derive an optimal criterion for the probability that a collided node will change its firing phase in the following next period after it acknowledges the collision.
- With the criterion, a method for a collided node to determine whether it changes its firing phase or not in a distributed manner is proposed.
- The performances of the proposed method are compared with existing Ext-Desync-based TDMA schemes and CSMA/CA.

The rest of the paper is organized as follows. In Section 2, overview Desync-TDMA and Ext-Desync. In Section 3, the problem that Ext-Desync schemes have in MANETs is illustrated. Then, the proposed method is explained. The numerical results are presented in Section 4. Studies that extend Desync-TDMA and Ext-Desync are described in Section 5. We also compare this work and the related work from a functional point of view in this section. Finally, the paper is concluded in Section 6.

## 2. Background

Here, an overview of the methods underlying the proposed method is illustrated. In Table 1, the main variables used for the illustration are listed.

### 2.1. Desync-TDMA

Desync-TDMA [10,11] is a decentralized and distributed TDMA protocol inspired by the firefly’s habits for single-hop wireless sensor nodes. In Desync TDMA, the time is managed by a constant cyclic period of *T*. Each node broadcasts a control packet (firingmessage) once at a predetermined time (firingphase) in every *T* cycle.

Let *N* and $\Phi_{i}\left(t\right)$ be the number of nodes and the firing phase of node *i* in the *t*-th *T* cycle, respectively, where i=1,2,⋯,N and t=1,2,⋯. Let $\Phi_{i,-}\left(t\right)$ and $\Phi_{i,+}\left(t\right)$ be the firing phases of other nodes just before and after $\Phi_{i}\left(t\right)$ in the *t*-th *T* cycle, respectively. Then, the firing phase of node *i* at the next (*t* + 1)-th *T* cycle, $\Phi_{i}\left(t+1\right)$, is calculated as

(1) $$\Phi_{i}\left(t+1\right)=T+\left(1-\alpha \right)\times \Phi_{i}\left(t\right)+\alpha \times \left(\Phi_{i,-}\left(t\right)+\Phi_{i,+}\left(t\right)\right)/2,$$

where α (0<α≤1) is a constant indicating how $\Phi_{i}\left(t+1\right)$ is closely calculated from the average of $\Phi_{i,-}\left(t\right)$ and $\Phi_{i,+}\left(t\right)$.

The slot that node *i* can transmit its data in (t+1)-th *T* cycle is calculated as follows.

(2) $$S_{i,st}\left(t+1\right)=T+\left(\Phi_{i,-}\left(t\right)+\Phi_{i}\left(t\right)\right)/2,$$

(3) $$S_{i,ed}\left(t+1\right)=T+\left(\Phi_{i}\left(t\right)+\Phi_{i,+}\left(t\right)\right)/2,$$

where $S_{i,st}\left(t+1\right)$ and $S_{i,ed}\left(t+1\right)$ denote the start and end times of the slot, respectively.

**Figure 1:**
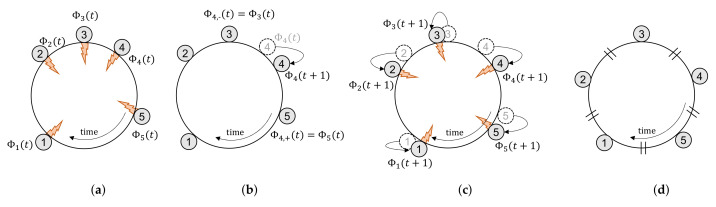
An example of the Desync-TDMA process for *N* = 5: (**a**) firings in the *t*-th period (**b**) node 4’s perspective in the *t*-th period (**c**) firings in (t+1)-th period, and (**d**) converged period.

Figure 1 shows an example of Desync-TDMA operation for *N* = 5. Figure 1a shows the firing phases of all nodes in the *t*-th period, $\Phi_{i}$ (i=1,2,⋯,5). Each node can calculate its firing phases and slot in the following (*t* + 1)-th period with the firing phases received from other nodes. Figure 1b shows the process of adjusting the firing phase in the following (t+1)-th period from the perspective of node 4. In this example, the firing phases immediately before and after $\Phi_{4}\left(t\right)$ are those by nodes 3 and 5, i.e., $\Phi_{4,-}\left(t\right)=\Phi_{3}\left(t\right)$ and $\Phi_{4,+}\left(t\right)=\Phi_{5}\left(t\right)$.

Then, node 4 calculates its firing phase and slot in the (t+1)-th period, $\Phi_{5}\left(t+1\right)$, $S_{5,st}\left(t+1\right)$ and $S_{5,ed}\left(t+1\right)$, respectively, using Equations (1)–(3). Similarly, other nodes also calculate their firing phases and slots in the (t+1)-th period, as shown in Figure 1c. As this process is repeated, it reaches the convergence state, as shown in Figure 1d, in which the firing phases and slots of nodes are evenly distributed within every T cycle.

### 2.2. Ext-Desync

Ext-Desync [25] is a multihop extension of Desync-TDMA. Ext-Desync solves the hidden terminal problem by making all nodes simply know all their two-hop neighbors and their relative firing times as follows: Node *i* in an Ext-Desync network has its own identifier ($a_{i}$) and maintains the set of its *h*-hop neighbors acknowledged in the *t*-th period as follows:

(4) $$L_{i}^{h}\left(t\right)=\left\{\left(a_{i,j}^{h}\left(t\right),\Delta_{i,j}^{h}\left(t\right)\right)|j=1,2,\ldots ,N_{i}^{h}\left(t\right)\right\},$$

where h=1,2, $N_{i}^{h}\left(t\right)$ denotes the number of *h*-hop neighbor nodes, and $a_{i,j}^{h}\left(t\right)$ and $\Delta_{i,j}^{h}\left(t\right)$ are the identifier and the relative firing phase with node *i* of *j*-th *h*-hop neighbor node, respectively. Node *i* can obtain $\Delta_{i,j}^{h}\left(t\right)$ as

(5) $$\Delta_{i,j}^{h}\left(t\right)=\Phi_{i,j}^{h}\left(t\right)-\Phi_{i}\left(t\right),$$

where $\Phi_{i,j}^{h}\left(t\right)$ denotes the firing phase of node $a_{i,j}^{h}\left(t\right)$. It is noted that $\left(a_{i},0\right)\in L_{i}^{1}\left(t\right)$ and $L_{i}^{1}\left(t\right)\subseteq L_{i}^{2}\left(t\right)$.

During *t*-th period, node *i* broadcasts a firing message containing $L_{i}^{1}\left(t-1\right)$ at its firing phase and receives firing messages from its 1-hop neighbor nodes. Then, $L_{i}^{1}\left(t\right)$ is updated by referring to the nodes’ firing messages that node *i* received. The nodes listed in the 1-hop neighbors’ firing messages, but not included in $L_{i}^{1}\left(t\right)$, become node *i*’s 2-hop neighbors, and they are updated in $L_{i}^{2}\left(t\right)$.

Let $\Delta_{i,-}\left(t\right)$ and $\Delta_{i,+}\left(t\right)$ be the relative firing phases just before and after 0 in $L_{i}^{2}\left(t\right)$. Then, the firing phase and the slot of node *i* at (t+1)-th period are determined by

(6) $$\Phi_{i}\left(t+1\right)=T+\Phi_{i}\left(t\right)+\alpha \times \left(\Delta_{i,-}\left(t\right)+\Delta_{i,+}\left(t\right)\right)/2,$$

(7) $$S_{i,st}\left(t+1\right)=T+\Phi_{i}\left(t\right)+\left(\Delta_{i,-}\left(t\right)+G\right)/2,$$

(8) $$S_{i,ed}\left(t+1\right)=T+\Phi_{i}\left(t\right)+\left(\Delta_{i,+}\left(t\right)-G\right)/2,$$

where *G* denotes the time-lag between adjacent slots for the firing message of a new node or a node changing its firing phase after collision not to violate slots of other nodes.

Due to the hidden terminal problem and the nodes’ movements, firing messages may collide with others. If a node does not know that the collision has occurred, the node continues to transmit packets in the next period, and it results in packet losses or retransmissions due to the losses, which degrades the packet delivery performances.

To solve the problem, Ext-Desync provides the method for node *i* to acknowledge the collision by monitoring the firing message from other nodes *j* within its one-hop range whether its identifier is not included in $L_{j}^{1}$ (j≠i). When the node recognizes the collision of its firing message, it may decide whether it changes its firing phase in the next period or not according to a certain probability (e.g., 0.5). The fixed probability-based decision policy may cause the degradation of networking performances, which are discussed in Section 3.1.

## 3. Extended-Desync TDMA with Optimal Criterion to Change the Firing Phase

### 3.1. Problem Definition

As mentioned in Section 2.2, when Ext-Desync is operated in MANET environments, collisions of firing messages may occur due to node mobility. Node *i* ($a_{i}$) can detect the collision of its firing message by monitoring the firing message from other node *j* within its one-hop range during the next periods whether its identifier is included in $L_{j}^{1}$ (j≠i).

**Figure 2:**
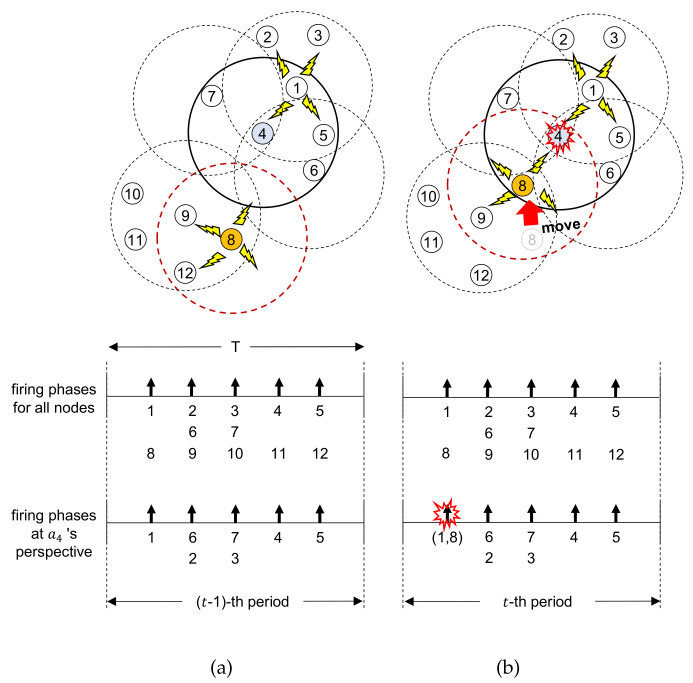
Example topology and timelines for firing messages: (**a**) before and (**b**) after $a_{8}$ moves.

Figure 2 shows an example scenario to illustrate this situation more clearly. There are 12 nodes denoted $a_{i}$ (*i* = 1,2,⋯,12), and $a_{8}$ is moving while other nodes are not. Figure 2a,b show the network topologies in (*t* − 1)-th and *t*-th periods before and after $a_{8}$ moves, respectively.

In Figure 2a, example topology and timelines of the firing messages from both all nodes and $a_{4}$’s perspective at (*t* − 1)-th period before $a_{8}$ moves are shown. Since nodes $a_{2}$, $a_{6}$, and $a_{9}$ are three-hop distances from each other, collision does not occur though they have the same firing phase [36,37]. Node $a_{4}$ has $L_{4}^{1}\left(t-1\right)=\left\{a_{1},a_{4},a_{5},a_{6},a_{7}\right\}$ and $L_{4}^{2}\left(t-1\right)=\left\{L_{4}^{1}\left(t-1\right),a_{2},a_{3}\right\}$.

When $a_{8}$ moves into the one-hop range of $a_{4}$ at *t*-th period as shown in Figure 2b, since $a_{8}$’s firing phase overlap with $a_{1}$’s one, firing messages from $a_{8}$ and $a_{1}$ are collided at $a_{4}$. $a_{4}$ makes its firing message $L_{4}^{1}\left(t\right)\neg ∋\left\{a_{1},a_{8}\right\}$. After $a_{8}$ and $a_{1}$ receive the firing message from $a_{4}$, they can acknowledge the collision of their firing messages.

**Figure 3:**
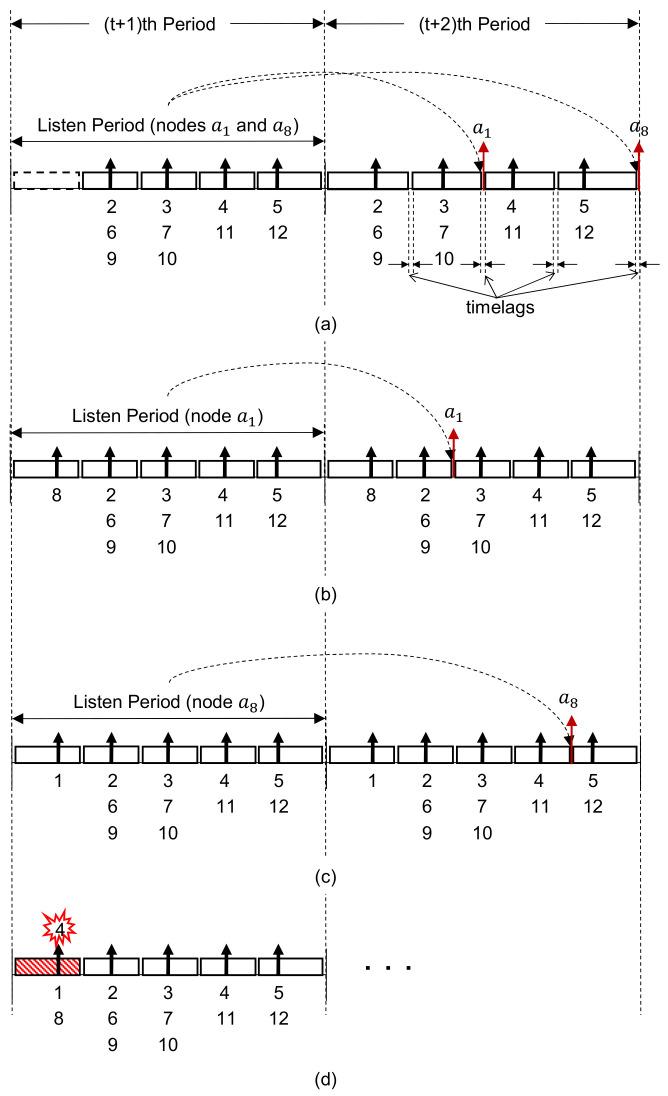
Example process to change the firing phases just after Figure 2b by (**a**) both $a_{1}$ and $a_{8}$, (**b**) $a_{1}$ only, (**c**) $a_{8}$ only, and (**d**) none.

After the collision is detected by $a_{1}$ and $a_{8}$ during *t*-th period, the possible cases where they can resolve the collision by changing their firing phases are shown in Figure 3. Figure 3a shows one case where both $a_{1}$ and $a_{8}$ change their firing phases. Nodes $a_{1}$ and $a_{8}$ do not transmit any messages, but listen to the firing messages from neighbors during (t+1)-th period. No node can utilize the slot allocated for $a_{1}$ and $a_{8}$ previously, which are denoted by the dotted line, and thus the slot is wasted. They calculate timelags, randomly choose one among them, and proceed with the join process to determine their slots in the network during (t+2)-th period as described in Section 2.2. Then, they can transmit data on their determined slots from (t+3)-th period.

Figure 3b shows the case where $a_{1}$ changes its firing phase while $a_{8}$ does not. In this case, $a_{8}$ can continuously utilize its slot previously assigned, no slot is wasted, unlike Figure 3a. However, after the listen and join processes, $a_{1}$ can transmit its data from (t+3)-th period. The case where only $a_{8}$ changes its firing phase is shown in Figure 3c. In this case, the opposite situation appears for $a_{1}$ and $a_{8}$ as in Figure 3b.

Figure 3d shows the case where both $a_{1}$ and $a_{8}$ do not change their firing phases. Then, since their firing messages collide again during (t+1)-th period, they should repeat one of Figure 3a–d from (t+2)-th period, and the slot is wasted.

### 3.2. Criterion of Firing Phase Change to Maximize Slot Utilization

The frequent change of firing phases due to collisions may cause significant degradations of Ext-Desync networking performances, especially from the viewpoints of the slot utilization as described in Section 3.1. The authors in [25] suggested that nodes that detect the collision may change their firing phases with a certain probability of 50% as a trade-off between reliability and latency. However, when the probability is fixed like that, there always exists the possibility that collided nodes change their firing phases simultaneously to equal phases, which results in collisions again and the waste of slots as shown in Figure 3.

In this Section, to overcome the problem by the fixed probability model as in [25], we derive an effective criterion model for collided nodes to determine the probabilities that they can change their firing phases dynamically by considering collision situations in order to maximize the slot utilization. The variables to explain the model are illustrated in Table 2.

For the convenience of the model derivation, it is assumed that $a_{i}$ acknowledges its firing message collision in the 0-th period, i.e., *t* = 0. As shown in Figure 3a, when $a_{i}$ changes its firing phase, since it can obtain the slot for data transmission in the following second period after the change of the firing phase, the slot is wasted for the periods. On the other hand, if $a_{i}$ does not change its firing phase, it can utilize the slot assigned to it continuously, as shown in Figure 3b,c.

Let $p_{c,i}\left(k\right)$ be the probability that $a_{i}$ changes its firing phase in the *k*-th period. $p_{nc,i}\left(k\right)=1-p_{c,i}\left(k\right)$. Then, the expected slot size for $a_{i}$ when it changes its firing phase before *n*-th period (n≥1) is written as

(9) $${\tilde{s}}_{c,i}^{*}\left(n\right)=\sum_{k=1}^{n}\left\{\left(n-k-2\right)s_{c,i}\left(k\right)p_{c,i}\left(k\right)\prod_{m=1}^{k}p_{nc,i}\left(m-1\right)\right\},$$

where $s_{c,i}\left(k\right)$ denotes the slot size for $a_{i}$, when it changes its firing phase in the *k*-th period and $p_{c,i}\left(0\right)$=0.

We also have the expected slot size for $a_{i}$ when it does not change its firing phase until the *n*-th period as

(10) $${\tilde{s}}_{nc,i}^{*}\left(n\right)=ns_{c,i}\left(0\right)\prod_{k=1}^{n}p_{nc,i}\left(k\right).$$

From Equations (9) and (10), the expected value of $s_{i}\left(n\right)$ is written as

(11) $$E\left[s_{i}\left(n\right)\right]={\tilde{s}}_{c,i}^{*}\left(n\right)+{\tilde{s}}_{nc,i}^{*}\left(n\right).$$

The collision may occur by the hidden terminal problem, for example, between $a_{1}$ and $a_{8}$ shown in Figure 2b. Since firing messages are broadcast, the collision of other nodes’ firing messages can be recognized by monitoring $L_{*}^{1}$ from others in the next period. Let ${\hat{C}}_{i}\left(n\right)$ be the number of those hidden nodes that cause the collision to $a_{i}$’s firing message in the *n*-th period. For example, nodes $a_{1}$ and $a_{8}$ are hidden terminals each other in Figure 2b. Accordingly, for $a_{1}$, we have ${\hat{C}}_{1}\left(0\right)$=1 and $c_{1,1}=a_{8}$. On the other hand, ${\hat{C}}_{8}\left(0\right)=1$ and $c_{8,1}=a_{1}$ for $a_{8}$. With ${\hat{C}}_{i}\left(n\right)$, the expected value of $s_{i,col}\left(n\right)$ can be calculated by

(12) $$E\left[s_{i,col}\left(n\right)\right]=\frac{{\hat{C}}_{i}\left(n\right)}{N_{i}^{1}\left(n\right)}{\tilde{p}}_{nc,i}^{*}\left(n\right).$$

${\hat{C}}_{i}\left(n\right)$s (n≥1) can be estimated consecutively using ${\hat{C}}_{i}\left(0\right)$ and the firing messages received from its one-hop neighbors in the *n*-th period as follows:

(13) C^i(n)=∑j=0C^i(n−1)C^i(n−1)j·j·{1−p^c,i(n)}j·p^c,i(n)C^i(n−1)−j.

Using Equations (11) and (12), the expected value of $s_{i,suc}\left(n\right)$ is calculated as

(14) $$E\left[s_{i,suc}\left(n\right)\right]=E\left[s_{i}\left(n\right)\right]-E\left[s_{i,col}\left(n\right)\right].$$

Assuming that $a_{i}$ acknowledges the collision of its firing message in the 0-th period and changes the firing phase with probability *p* (0≤p≤1) in the next 1-st period, let define ${\bar{ES}}_{i}\left(p,n\right)$ as the expected amount of total data successfully transmitted up to the *n*-th period. With Equations (11) to (14), it can be written as

(15) $$\begin{array}{cc} {\bar{ES}}_{i}\left(p,n\right) & =\sum_{k=1}^{n}E\left[s_{i,suc}\left(k\right)\right]\\ \; & =\left(n-1\right)+{\left(1-p\right)}^{n}-\sum_{k=1}^{n}CN_{i}\left(k\right){\left(1-p\right)}^{k}, \end{array}$$

where $p=p_{c,i}\left(1\right)$ and $CN_{i}\left(k\right)={\hat{C}}_{i}\left(k\right)/N_{i}^{1}\left(k\right)$.

Then, we have the criterion to determine the probability that $a_{i}$ changes its firing phase to maximize ${\bar{ES}}_{i}\left(p,n\right)$ as following:

**Proposition** **1.** *When $a_{i}$ acknowledges first the collision of its firing message in a period, ${\bar{ES}}_{i}\left(p,n\right)$ is maximized when it changes its firing phase in the next period with the probability of $p_{c,i}\left(1\right)$ = 0 or 1.*

**Proof** **of Proposition 1.** From Equation (15), we have ${\bar{ES}}_{i}\left(0,n\right)$ and ${\bar{ES}}_{i}\left(1,n\right)$ when $p_{c,i}\left(1\right)$ = 0 and 1, respectively, as follows

(16) $${\bar{ES}}_{i}\left(0,n\right)=n-\sum_{k=1}^{n}CN_{i}\left(k\right),$$

(17) $${\bar{ES}}_{i}\left(1,n\right)=n-1.$$

To validate the Proposition that ${\bar{ES}}_{i}\left(0,n\right)$ or ${\bar{ES}}_{i}\left(1,n\right)$ are always greater than ${\bar{ES}}_{i}\left(p^{\prime },n\right)$ for $0<p^{\prime }<1$, we consider the following two cases.

Case 1. ${\bar{ES}}_{i}\left(0,n\right)>{\bar{ES}}_{i}\left(1,n\right)$:

In this case, from Equations (16) and (17), we have $\sum_{k=1}^{n}CN_{i}\left(k\right)<1$. For $0<p^{\prime }<1$, the inequity of $\sum_{k=1}^{n}CN_{i}\left(k\right)\left\{1-{\left(1-p^{\prime }\right)}^{k}\right\}<\left\{1-{\left(1-p^{\prime }\right)}^{n}\right\}$ can be obtained. With the inequalities and Equation (15), we have the relationship between ${\bar{ES}}_{i}\left(0,n\right)$ and ${\bar{ES}}_{i}\left(p^{\prime },n\right)$ as follows

(18) $$\begin{array}{cc} {\bar{ES}}_{i}\left(0,n\right) & -{\bar{ES}}_{i}\left(p^{\prime },n\right)\\ \; & =\left\{n-\sum_{k=1}^{n}CN_{i}\left(k\right)\right\}-\left\{\left(n-1\right)+{\left(1-p^{\prime }\right)}^{n}-\sum_{k=1}^{n}CN_{i}\left(k\right){\left(1-p^{\prime }\right)}^{k}\right\}\\ \; & =1-{\left(1-p^{\prime }\right)}^{n}-\sum_{k=1}^{n}CN_{i}\left(k\right)\left\{1-{\left(1-p^{\prime }\right)}^{k}\right\}>0 \end{array}$$

Case 2. ${\bar{ES}}_{i}\left(0,n\right)\leq {\bar{ES}}_{i}\left(1,n\right)$:

This case is equivalent to $\sum_{k=1}^{n}CN_{i}\left(k\right)\geq 1$. For $0<p^{\prime }<1$, we have $\sum_{l=1}^{n}CN_{i}\left(k\right){\left(1-p^{\prime }\right)}^{k}>{\left(1-p^{\prime }\right)}^{n}$. With the inequalities and Equation (15), the following relationship between ${\bar{ES}}_{i}\left(1,n\right)$ and ${\bar{ES}}_{i}\left(p^{\prime },n\right)$ holds for this case.

(19) $$\begin{array}{cc} {\bar{ES}}_{i}\left(1,n\right) & -{\bar{ES}}_{i}\left(p^{\prime },n\right)\\ \; & =\left(n-1\right)-\{\left(n-1\right)+{\left(1-p^{\prime }\right)}^{n}-\sum_{k=1}^{n}CN_{i}\left(t,k\right){\left(1-p^{\prime }\right)}^{k}\}\\ \; & =-{\left(1-p^{\prime }\right)}^{n}+\sum_{k=1}^{n}CN_{i}\left(t,k\right)\left\{1-{\left(1-p^{\prime }\right)}^{k}\right\}>0 \end{array}$$

Cases 1 and 2 indicate that either of ${\bar{ES}}_{i}\left(0,n\right)$ or ${\bar{ES}}_{i}\left(1,n\right)$ is always larger than ${\bar{ES}}_{i}\left(p^{\prime },n\right)$ for $0<p^{\prime }<1$. In other words, ${\bar{ES}}_{i}\left(p,n\right)$ is maximized when $a_{i}$ changes its firing phase with the probability of 0 or 1, not determining probabilistically between 0 and 1. □

**Figure 4:**
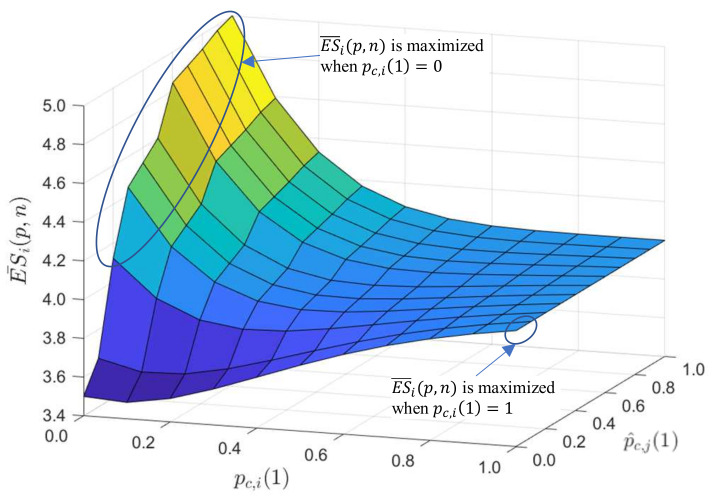
An example results of ${\bar{ES}}_{i}\left(p,n\right)$ with varying $p_{c,i}\left(1\right)$ and ${\hat{p}}_{c,j}\left(1\right)$ (when n=5, $N_{i}^{1}\left(0\right)=10$, and ${\hat{C}}_{i}\left(0\right)=3$).

To show the effectiveness of the Proposition, the values of ${\bar{ES}}_{i}\left(p,n\right)$ for various $p_{c,i}\left(1\right)$ and ${\hat{p}}_{c,j}\left(1\right)$ when *n* = 5 and $N_{i}^{1}\left(0\right)$ = 10 are shown in Figure 4. For the convenience, it is assumed that the slot size of each node is set to 1, i.e., $s_{c,i}\left(k\right)$ = 1, for ∀i and ∀k. As shown in Figure 4, ${\bar{ES}}_{i}\left(p,n\right)$ shows different aspects for each $p_{c,i}\left(1\right)$ and ${\hat{p}}_{c,j}\left(1\right)$ and is maximized when $p_{c,i}\left(1\right)$ is 1 or 0, and it appears differently depending on ${\hat{p}}_{c,j}\left(1\right)$.

That is, when ${\hat{p}}_{c,j}\left(1\right)$s are lower than around 0.2, ${\bar{ES}}_{i}\left(p,n\right)$s show the increasing pattern as $p_{c,i}\left(1\right)$ increases, and thus ${\bar{ES}}_{i}\left(p,n\right)$s become maximum at $p_{c,i}\left(1\right)$ = 1. On the other hand, when ${\hat{p}}_{c,j}\left(1\right)$s are greater than around 0.2, ${\bar{ES}}_{i}\left(p,n\right)$s show a decreasing pattern as ${\hat{p}}_{c,j}\left(1\right)$ increases, which results in the maximums of ${\hat{p}}_{c,j}\left(t,1\right)$s at $p_{c,i}\left(1\right)$ = 0. Likewise, ES becomes its maximum at $p_{c,i}\left(t,1\right)$ = 0 or 1 depending on ${\hat{p}}_{c,j}\left(1\right)$. As mentioned earlier, it is not possible for $a_{i}$ to know ${\hat{p}}_{c,j}\left(1\right)$. The method to estimate ${\hat{p}}_{c,j}\left(1\right)$ is explained in Section 3.3 as one of our proposed methods in this paper.

With the Proposition, we have the optimal criterion to determine the probability for changing its firing phase as follows

(20) $$p_{c,i}\left(1\right)=\left\{\begin{array}{cc} 0, & {\bar{ES}}_{i}\left(0,n\right)>{\bar{ES}}_{i}\left(1,n\right)\\ 1, & otherwise \end{array}\right.$$

### 3.3. Ext-Desync TDMA with Optimal Criterion of Firing Phase Change

**Figure 5:**
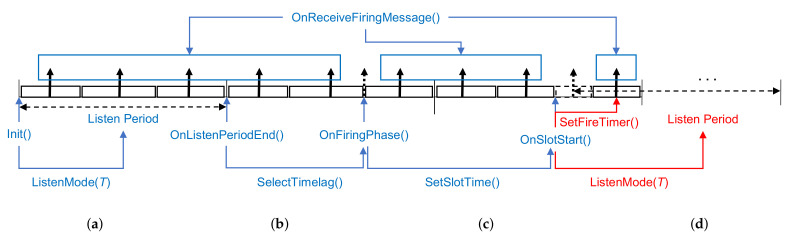
An example of the Proposed Ext-Desync process from the standpoint of the main functions: (**a**) 0th period (**b**) 1st period (**c**) 2nd period (**d**) after 2nd period.

Here, algorithms for determining the optimal criterion of firing phase change illustrated in the previous section are explained. For better understanding, the relationships among the functions used in the algorithms and their operational processes concerning cycle periods are shown in Figure 5. Since the proposed method operates asynchronously, like Ext-Desync, the algorithms also operate asynchronously. A detailed explanation of the operation is explained later. ListenMode(T) in Algorithm 1 is executed every period, and The algorithm’s operation starts from ListenMode(T) in Algorithm 1 every period by receiving firing messages from nodes and detecting whether its firing message collided or not.

The period executing ListenMode(T) is denoted as the 0-th period for convenience. The blue line and text in Figure 5 are the main functions used in Algorithm 1 showing their interrelationship on operations according to subsequent periods following the 0-th period. Among Algorithm 1 functions, OnSlotStart( ) operation depends on the result of DeterminePhaseChange( ) function performed in Algorithm 2. The red line and text indicate the process of Algorithm 1 according to the result of Algorithm 2.

The overall procedure of Ext-Desync TDMA with the adaptive change of the firing phase from the viewpoints of a node $a_{i}$ (i=1,2,⋯) in the network is shown in Algorithm 1 with five main functions such as Init(), OnListenPeriodEnd(), OnReceive- FiringMessage(), OnFiringPhase(), and OnSlotStart(). Since Algorithm 1 operates repeatedly, the period index is expressed to 0. Notations of •(0) and •(1) refer to the parameters of the current and the following periods, respectively.

Init() is done when $a_{i}$ is initially joining the network and starts to execute the adaptive firing phase change mode.

OnListenPeriodEnd() is called at the end of the listen period, i.e., at the end of ListenMode() in Init(), which is mentioned in Section 3.1. ${\hat{p}}_{c,i}\left(1\right)$ is initialized by ${\hat{p}}_{c,0}$, which is one of the system parameters and used in DeterminePhaseChange() of Algorithm 2. The firing phase in the following period, $\Phi_{i}\left(1\right)$, is randomly selected by SelectTimelag() as illustrated in Figure 3.

**Algorithm 1:** Proposed Ext-Desync TDMA Procedure with Optimal Criterion of Firing Phase Change.

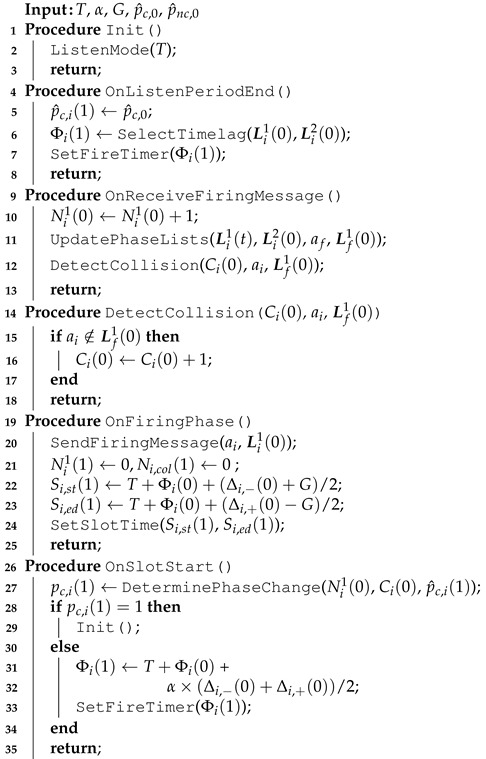



OnReceiveFiringMessage() is called whenever $a_{i}$ receives a firing message from an one-hop neighbor denoted by $a_{f}$ with $L_{f}^{1}\left(0\right)$. Then, $N_{i}^{1}\left(0\right)$ is increased by one, and $L_{i}^{1}\left(0\right)$ and $L_{i}^{2}\left(0\right)$ are updated using $a_{f}$ and $L_{f}^{1}\left(0\right)$ by UpdatePhaseLists(). To update $C_{i}\left(0\right)$, it calls DetectCollision(), in which it is increased by one when $a_{i}$ is not included in $L_{f}^{1}\left(0\right)$.

In OnFiringPhase(), $a_{i}$ sends its firing message, initializes $N_{i}^{1}$ and $C_{i}$ to 0. Then, it calculates and allocates its slot in the following period using Equations (7) and (8).

OnSlotStart() is invoked at the slot start time set by SetSlotTime( ). According to the decision on $p_{c,i}\left(1\right)$ by DeterminePhaseChange(), which is explained in Algorithm 2, it enters the listen period to reconnect with other devices in the network if $p_{c,i}\left(1\right)$ is 1, as shown in Figure 3 in Section 3.1. On the other hand, it sets the next firing phase.

**Algorithm 2:** Determination of Firing Phase Change with Optimal Criterion.

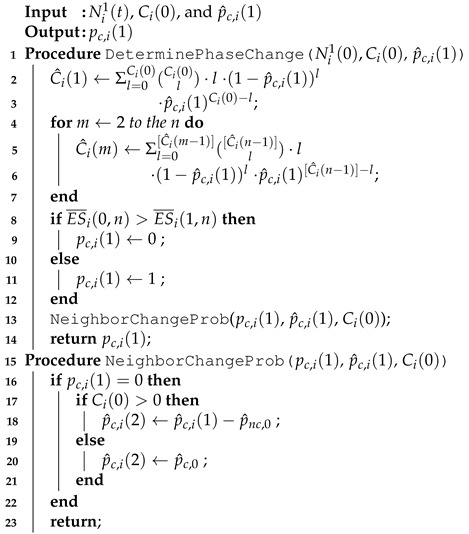



Algorithm 2 shows the procedures to determine whether to change the firing phase in the following period or not. It utilizes $N_{i}^{1}\left(0\right)$, $C_{i}\left(0\right)$, and ${\hat{p}}_{c,i}\left(1\right)$ obtained from OnSlotStart() in Algorithm 1. The purpose of Algorithm 2 is to derive the probability for changing the firing phase, $p_{c,i}\left(1\right)$. Accordingly, we can have the probability of $p_{c,i}\left(1\right)$ as the result of Algorithm 2.

In DeterminePhaseChange(), it computes ${\hat{C}}_{i}\left(n\right)$ (n≥1) sequentially using Equation (13). From the Proposition and Equation (20), $p_{c,i}\left(1\right)$ is determined. Then, it calls Neighbor Change- Prob() to update ${\hat{p}}_{c,i}\left(2\right)$ using ${\hat{p}}_{c,i}\left(1\right)$.

In NeighborChangeProb(), $a_{i}$ calculates ${\hat{p}}_{c,i}\left(2\right)$ based on ${\hat{p}}_{c,i}\left(1\right)$. When $p_{c,i}\left(1\right)=1$, $a_{i}$ will enter the listen mode in the following period by setting ${\hat{p}}_{c,i}\left(2\right)$ to ${\hat{p}}_{c,0}$. Thus, no further action for this case is needed in NeighborChangeProb(). The case where $p_{c,i}\left(1\right)=0$ and $C_{i}\left(0\right)>0$ means that the neighbors caused the collision will not change their firing phases. Then, ${\hat{p}}_{c,0}\left(2\right)$ is set to ${\hat{p}}_{c,0}\left(1\right)-{\hat{p}}_{nc,0}$, where ${\hat{p}}_{nc,0}$ denotes the initialization parameter of ${\hat{p}}_{nc,i}\left(1\right)$, which are determined as the system operational parameters by the administrator. On the other hand, i.e., if $C_{i}\left(0\right)$ = 0, ${\hat{p}}_{c,0}\left(t+1\right)$ is set to ${\hat{p}}_{c,0}$ since no collision occurred. It is noted that ${\hat{p}}_{c,i}\left(n\right)$s, for n=3,⋯, are calculated at the next period in the same way.

Since the proposed method follows the same process of Ext-Desync, the complexity for running time of the proposed method is the same as that for Ext-Desync. Therefore, it becomes $O({\left({\bar{N}}_{1}+{\bar{N}}_{2}\right)}^{2})$ according to [10], where ${\bar{N}}^{1}$ and ${\bar{N}}^{2}$ are the average number of one-hop and two-hop neighbors, respectively.

## 4. Numerical Results

The performances of the proposed method (*Proposed*) are compared to those of the following existing schemes: *CSMA/CA*, *Ext-Desync* with the phase changing probability of 50% fixed as illustrated in Section 2.2 [25] and the adjustment of the Ext-Desync scheme proposed in [35] (*Adjustment*) in which the firing phase change probability is adjusted according to the network environment. We also compared the method to support multi-hop communications by extending Desync-TDMA, called Multi-hop Desync-TDMA (*MH-Desync*) [29]. The numbers of control and data time slots for *MH-Desync* were set to 40 and 80, respectively. We implemented the network simulator for the performance comparisons using the Riverbed Modeler (formerly, OPNET Modeler) [38].

We consider a MANET environment with 50 nodes moving in a 1000 × 1000 m region. The wireless link bandwidth and each node’s transmission range are set to 2 Mbps and 250 m, respectively. The random waypoint model is considered to model the mobile behaviors of the nodes. The mobility model was implemented in the Riverbed Modeler. The maximum movement speed of the node is set to 10, 20, and 30 m/s, which reflects the low, medium, and high-moving speeds, respectively.

Each simulation is carried out during 500 s for a given condition and repeatedly done over 10 times with different random seeds. As parameters for the proposed algorithm, ${\hat{P}}_{i}$ is set to 0.5. In addition, ${\hat{p}}_{nc,0}$ is set to 0.1.

### 4.1. Collision Resolution Performances

As mentioned in Section 2.2, in Ext-Desync, each node determines its slot to be used for data transmission every period considering the firing phases of 1- and 2-hop neighbors. The neighbor relationship may change according to nodes’ movements, and hence the slot size for a node may vary. The slots may be wasted due to collisions of firing messages, as illustrated in Figure 3.

**Figure 6:**
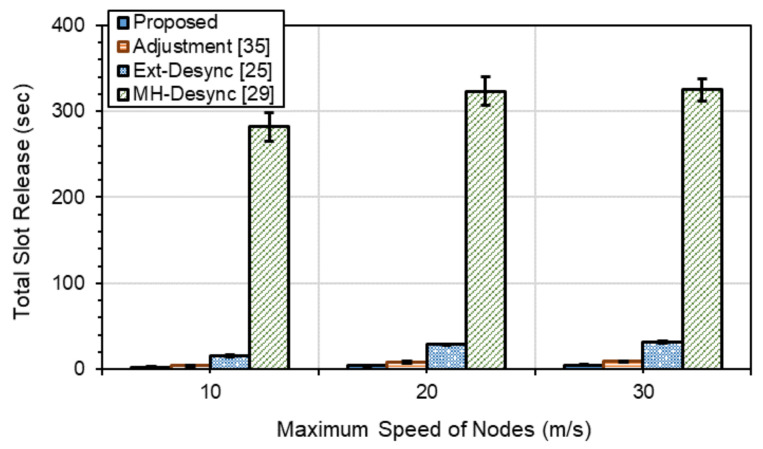
Total slots wasted due to collisions.

Figure 6 shows the total slots wasted by collisions of firing messages due to nodes’ mobility during a 500s simulation time. As the nodes’ maximum speeds increase, the possibility of firing messages’ collisions also increases, which increases wasted slots. As we can see from Figure 6, the *Proposed* and *Adjustment* methods have very few wasted slots and very small increments with increasing the node’s maximum speed, while, for *Ext-Desync*, this increases significantly. *MH-Desync* shows much higher wasted slots than others. Since the control and data channels are divided into fixed slots in *MH-Desync*, the control-slot and firing-phase information in the control channel may collide while nodes are moving, which results in much higher slot wastes.

The results of Figure 6 imply the performances on the degree of how quickly the methods can resolve the collision situation, and the proposed method shows the best. On average, slot waste of *Proposed* is 50%, 14.6% and 1.2% of the slot waste of *Adjustment*, *Ext-Desync* and *MH-Desync*, respectively.

**Figure 7:**
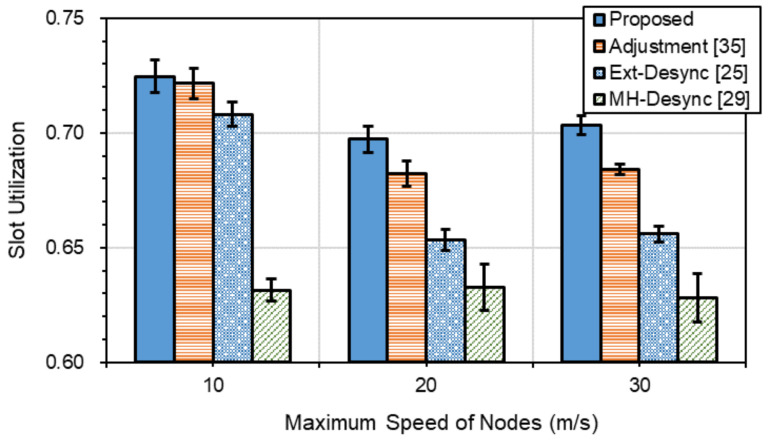
Average slot utilization.

Figure 7 depicts the slot utilization performance, which is defined as the ratio of the sum of slot sizes that have successfully transmitted data out of the total size of allocated slots to nodes. The less the wasted slots are, the more the slots available for data transmission are. *Proposed* with the least wasted slots shows the best slot utilization performance, while *MH-Desync* shows the worst. *Proposed* shows better slot utilization performances of 1.8%, 5.4% and 12.3% in average than *Adjustment*, *Ext-Desync* and *MH-Desync*, respectively.

### 4.2. Packet Delivery Performances

To compare packet delivery performances, the Ad hoc On-demand Distance Vector (AODV) routing protocol is considered. For packet generation, traffic flows are randomly paired among nodes, where we let *K* be the number of traffic flows. The packet size and the packet inter-arrival times are set constant at 356 bits and 0.03 s, respectively.

**Figure 8:**
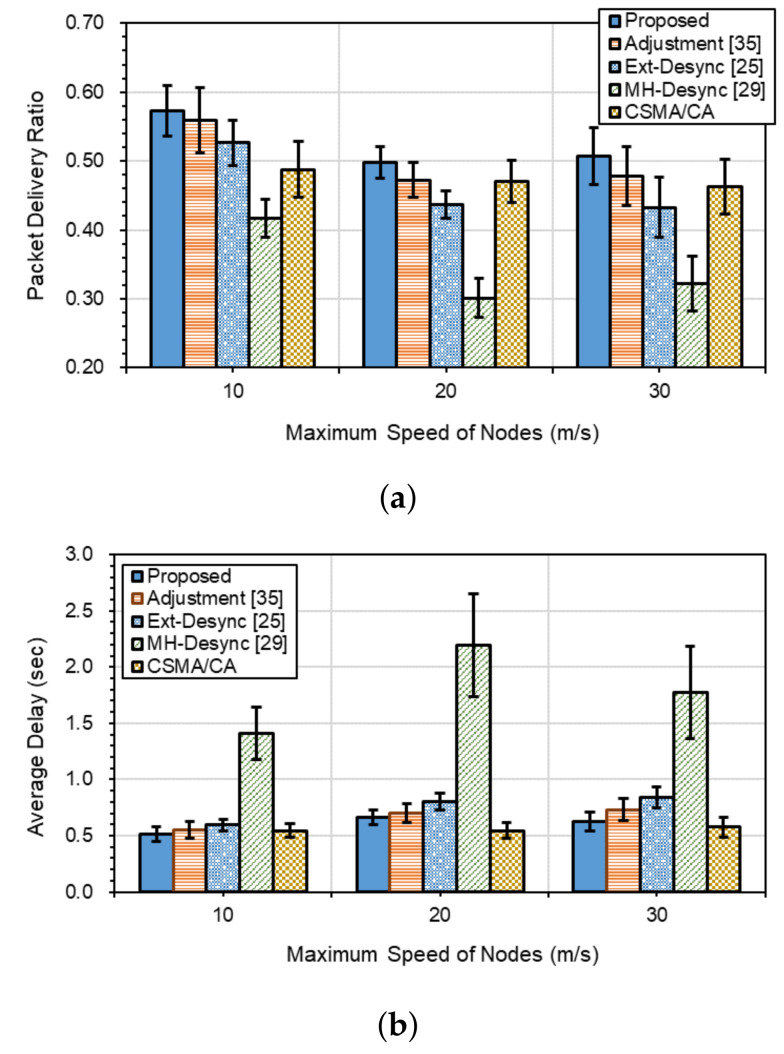
Packet delivery performances for *K* = 15: (**a**) packet delivery ratio, and (**b**) end-to-end delay.

Figure 8a,b show the packet delivery performances in terms of the packet delivery ratio (PDR) and the end-to-end delay (E2ED), respectively, when the number of traffic flows (*K*) is 15. As the nodes’ maximum speeds increase, PDRs decrease while E2EDs increases for all methods. Among the Ext-Desync-based schemes, as the maximum speed increases, *Proposed* shows the best, *Adjustment* and *Ext-Desync* the next, and *MH-Desync* the lowest in both PDR and E2ED performances.

*Adjustment*, *Ext-Desync*, *MH-Desync* and *CSMA/CD* show lower PDR performances of 4.5%, 13.1%, 51.7%, and 11% on average, respectively, than *Proposed*. The delay of *Proposed* is 9.4%, 19.4%, and 66.5% lower on average than *Adjustment*, *Ext-Desync*, and *MH-Desync*, respectively. On the other hand, *Proposed* shows 8.4% higher delay than *CSMA/CD*. The reason for the results can be derived from those shown in Figure 6. That is, as the maximum speed increases, the collision probability of firing messages increases, and times to resolve the collision situation are that *Proposed* is the shortest, *Adjustment* and *Ext-Desync* is the next, and *MH-Desync* is the longest.

On the other hand, PDRs and E2EDs for *CSMA/CD* are not significantly affected by the increase in maximum speed. As shown in Figure 6, *MH-Desync* shows a high amount of collisions when nodes move. Therefore, it is analyzed that data cannot be transmitted in time and has a very high delay than others. Particularly, as the maximum speed increases, PDRs of *CSMA/CD* are similar to Adjustment and show better than *Ext-Desync*. While, E2EDs of *CSMA/CD* show the best when the maximum speed is over 20 m/s. This is because *CSMA/CD* is a random access-based protocol and can transmit packets immediately as soon as it occupies the channel. Whereas, other methods can transmit packets only within slots allocated based on TDMA.

**Figure 9:**
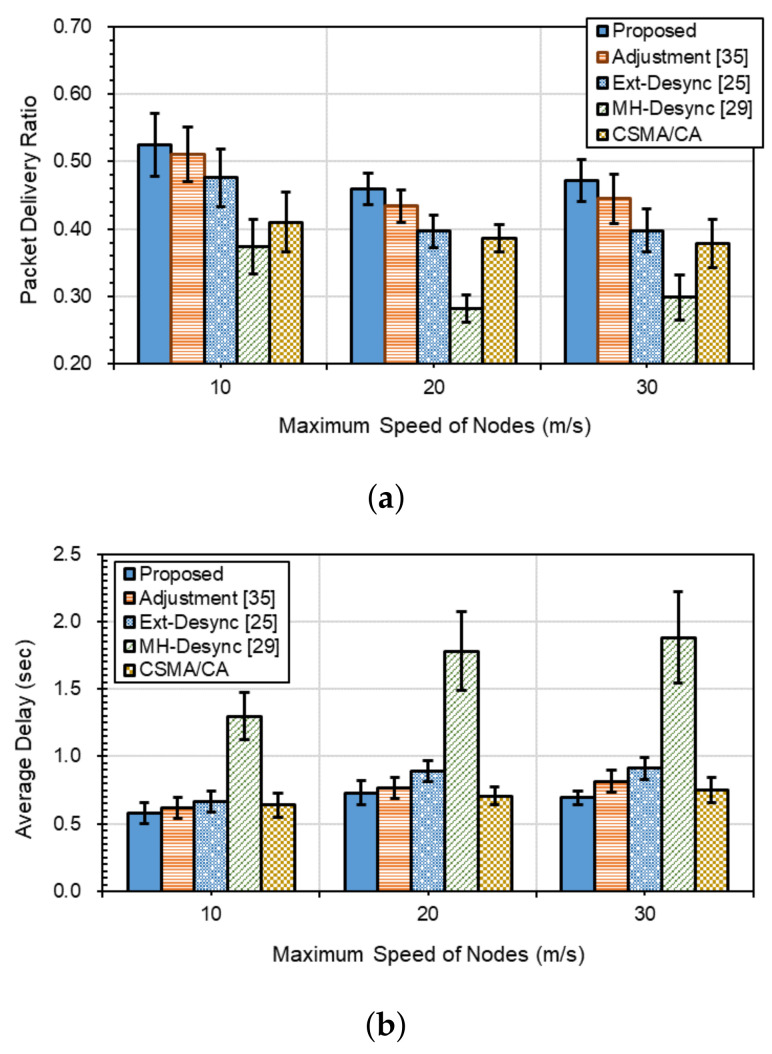
Packet delivery performances for *K* = 20: (**a**) packet delivery ratio, and (**b**) end-to-end delay.

When *K* is 20, compared with Figure 8 for *K* = 15, PDRs decrease while E2EDs increase for all methods, as shown in Figure 9a,b, respectively. As we can see from Figure 9a, PDR performances show the best in *Proposed*, followed by *Adjustment*, *Ext-Desync*, *MH-Desync* and *CSMA/CD*. *Adjustment*, *Ext-Desync*, *MH-Desync* and *CSMA/CD* show average 4.7%, 14.6%, 52.5% and 23.9%, respectively, lower PDR performances than *Proposed*. Unlike the case when *K* = 15 shown in Figure 8a, *CSMA/CD* show lower PDR than *Proposed*, *Adjustment*, and *Ext-Desync*. This is because *CSMA/CD* is a contention-based protocol.

That is, as *K* increases, the number of packets transmitted increases, and the degree of the contention to occupy the channel becomes serious. Likewise, as the traffic increases, Ext-Desync-based schemes show better PDRs than *CSMA/CD*. For both low and heavy traffic flows, *Proposed* shows the best. As shown in Figure 9b, *Proposed* shows the best E2EDs at all maximum speeds: the delays of *Adjustment*, *Ext-Desync*, *MH-Desync* and *CSMA/CD* are, respectively, 9.6%, 23.2%, 147.6%, and 4.5% on average higher than *Proposed*. While, as the maximum speed increases over 20 m/s, *CSMA/CD* outperforms *Adjustment* and *Ext-Desync* in E2ED. On the other hand, though *Proposed* is based on TDMA, it shows similar or better E2EDs with *CSMA/CA* even for high-speed moving environments.

## 5. Related Work and Discussion

In Desync-TDMA, all nodes periodically broadcast their firing messages every period. The firing time and the slot for each node to be utilized in the next period can be determined using the information included in firing messages from neighbors and their receiving times. The detailed process on the slot allocation is described in Section 2. The slot scheduling process is performed in a fully distributed way. After a certain period, the slot allocation situation converges if the network topology has not changed.

Desync-TDMA does not require global slot synchronization schemes as in ordinary TDMA-based methods [23,24]. Some extensions of Desync-TDMA [12,13,14,15,16,18,19,20,21,22] propose algorithm to cope with packet loss in lossy networks [12], to reduce desynchronization errors and convergence time [13,14,16,20,22], to provide weighted slot scheduling according to traffic demand of each node [15,17], or to reduce energy consumption [21]. The authors in [18] proposed the firing offset adjustment scheme to overcome the message split due to the firing message within a slot. In particular, Kuramoto-Desync [19] has all of the above advantages. However, they are not suitable for applying to MANET because they do not support the multi-hop MAC function.

In order to support multi-hop MAC function, in the Extended-Desync TDMA [25], the firing message transmitted by each node includes firing phase-related information of one-hop neighbors. However, when collisions of firing message are occurred due to the movement of the nodes, the nodes that caused the collisions change their firing phases based on the fixed probability without considering the about the surrounding environment. Therefore, as the maximum speed of the nodes increases, the performance of the Extended-Desync TDMA is extremely decreased due to the frequent change of the firing phases.

Decentralized round-robin and proportional fair scheduling [26] reduces desynchronization errors and convergence time of the Ext-Desync. MD-MAC [27], MH-Desync TDMA [29], and MH-PCO-D [34] are extensions of [25] with frame structure. These show better throughput than existing MAC protocols. Weighted-Desync TDMA [30] is an extension of [29] that provides weighted slot scheduling. However, due to the fixed frame structure, [27,29,30,34] require an additional global slot synchronization scheme to use in MANET.

Self-organizing transmission scheduling is a MAC protocol for efficiently transferring data from sensor nodes to a BS (Base Station). Each sensor node propagates its own hop count from the BS and uses schemes to avoid collisions with nodes having the same hop counts. However, it is difficult to use for MANET that can be operated in an environment in which there are a plurality of BSs or even each sensor node determines the situation by itself without the BS. Decentralized round-robin and proportional fair scheduling [26] provide weighted slot scheduling using two firing messages (start and end beacons).

The researchers in [28] proved that [26] provides MAC function without message collision even in locally connected networks. PulseSS [31] is a multi-hop extension of [26] to target an environment where at least one CH (Cluster Head) exists within the transmission range of each node. However, dynamic CH selection algorithm according to the network topology is additionally required.

In [32], the cross-layer approach in conjunction with Ext-Desync and ad-hoc routing protocols was proposed. In [33], a call admission control scheme was proposed to support QoS in an Ext-Desync-based MANET environment. In the Extended-Desync TDMA with adjustment of firing phase changing probability [35], each node adjusts its firing phase changing probability in every period based on the collision of firing messages occurred in the neighbors.

Table 3 shows the comparisons for representative Desync-TDMA and Ext-Desync-based proposals in terms of some essential networking features. Multi-hop support is one of the most important key components in distributed networking. Desync-TDMA-based methods focus on a single-hop Wireless Sensor Network (WSN) environment and do not support multi-hop. On the other hand, Ext-Desync-based ones support multi-hop communication as described in Section 2.2.

In multi-hop communication environments, collisions may occur due to the hidden terminal problem depending on the addition or mobility of nodes. Most of the methods supporting multi-hop communication provide ways to resolve collisions by the hidden terminal problem due to the addition of nodes. Still, they consider the situation in which the nodes are fixed. On the other hand, the proposed method and [35] support node mobility together.

Most Desync-TDMA and Ext-Desync-based methods focus on speeding up the convergence time for evenly distributed or weighted slot usages. However, as we raised in Section 3.1, the degradation effect of convergence time, packet loss, and slot utilization by firing message collision is not considered. Only the proposed method and [35] consider the firing message collision problem. In addition, only the proposed method utilizes the optimal criterion of firing phase change scheme, then provides the capability of quickly recognizing conflict situations and responding to conflict resolution faster than other ones. As a result, experiments showed that we could achieve performance improvements in convergence time, packet loss, and slot utilization.

In addition, most of the Desync-TDMA and Ext-Desync-based methods do not require global time synchronization. This means that Desync-TDMA and Ext-Desync-based methods are very effective ones that can utilize the advantages of TDMA fully without implying the global time synchronization problem, which is the critical limitation of TDMA-based methods.

## 6. Conclusions

In this paper, we dealt with the potential problem that Ext-Desync-based schemes have when operated in MANETs, which has been overlooked in other studies. Then, we derived a mathematical model to evaluate the problem. With the derived model, we proposed a method for a collided node to determine optimally whether it changes the firing phase or not in MANET. The proposed method enables collided nodes optimally to determine their phase changes in a distributed manner by considering both the collision situation and the slot utilization. In this way, the collision situation can be resolved effectively.

We showed the proposed method could resolve the collision situations much faster than other Ext-Desync-based schemes. The performances of the proposed method have been compared with CSMA/CA and other existing Ext-Desync-based schemes in terms of PDRs and E2EDs. Since Ext-Desync-based schemes are based on TDMA while CSMA/CA is random access and contention-based protocol, there have been trade-offs in PDRs and E2EDs among those schemes according to the variations of node moving speeds and the number of traffic flows. It was shown that, in all cases, the proposed method outperformed other comparable methods, including CSMA/CA.

TDMA-based approaches are particularly preferred in multihop tactical networking since TDMA provides dedicated network channels for users without contention while sharing network capacity. With the adjustment of dedicated channel sizes, this can also support Quality of Services (QoSs) prioritization.

For TDMA operations, time synchronization among TDMA nodes should be required. However, in tactical MANET environments without infrastructure support, global time synchronization support is limited. As mentioned before, Ext-Desync-based methods are effective in utilizing the advantages of TDMA fully without the global time synchronization problem. In particular, the proposed method is effectively applicable to the MANET environment by providing a fast recovery in firing message collision situations caused by moving nodes, which are not considered in other studies.

This is expected to support the differentiated traffic transmission with different QoS requirements by integrating the proposed method with existing works studied on QoS issues in Desync-TDMA based networks. Additionally, in tactical networking environments, the firing messages may be lost at the physical layer by jammers. It is expected that it will be possible to distinguish the collision situations by jamming or simultaneous transmissions by extending the collision monitoring process in Ext-Desync and the proposed method. We will study these issues in future works.

## Figures and Tables

**Table 1:** Variables for the illustration of Desync-TDMA and Ext-Desync.

Variable	Description
*T*	The cyclic period of Desync- and Ext-Desync-based schemes
*N*	The number of nodes in tne network
$\Phi_{i}\left(t\right)$	The firing phase of node *i* in *t*-th T cycle
$\Phi_{i,-}\left(t\right)$	The firing phase of other node just before $\Phi_{i}\left(t\right)$
$\Phi_{i,+}\left(t\right)$	The firing phase of other node just after $\Phi_{i}\left(t\right)$
$S_{i,st}\left(t\right)$	The start time of the node *i* in *t*-th period
$S_{i,ed}\left(t\right)$	The end time of the node *i* in *t*-th period
α	constant indicating how $\Phi_{i}\left(t+1\right)$ is calculated from the average of $\Phi_{i,-}\left(t\right)$ and $\Phi_{i,+}\left(t\right)$
$L_{i}^{h}\left(t\right)$	The list of firing phase information of *h*-hop neighbors managed by node *i* in the *t*-th period
$a_{i,j}^{h}\left(t\right)$	Identifier of *j*-th *h*-hop neighbor node of node *i* in *t*-th period
$\Delta_{i,j}^{h}\left(t\right)$	The relative firing phase with node *i* of *j*-th *h*-hop neighbor node in *t*-th period
$N_{i}^{h}\left(t\right)$	The number of *h*-hop neighbor node of node *i* in *t*-th period
$\Phi_{i,j}^{h}\left(t\right)$	The firing phase of node $a_{i,j}^{h}\left(t\right)$
*G*	The length of the timelag of Ext-Desync-based schemes

**Table 2:** Description of variables.

Variable	Description
$a_{i}$	Node that acknowledged the collision of its firing message in the 0-th period
$N_{i}^{1}\left(n\right)$	Number of one-hop neighbors of $a_{i}$ in the *n*-th period (n≥0)
${\hat{C}}_{i}\left(n\right)$	Number of hidden nodes that cause the collision to $a_{i}$’s firing message in the *n*-th period (n≥0)
$c_{i,j}\left(n\right)$	Nodes of hidden to $a_{i}$ in the *n*-th period ($j=1,2,\cdot ,{\hat{C}}_{i}\left(n\right)$)
$p_{c,i}\left(n\right)$	Probability that $a_{i}$ *changes* its firing phase in the *n*-th period (n≥0)
$p_{nc,i}\left(n\right)$	Probability that $a_{i}$ *does not change* its firing phase until the *n*-th period ($p_{nc,i}\left(n\right)=1-p_{c,i}\left(n\right))$ (n≥0)
${\tilde{s}}_{c,i}^{*}\left(n\right)$	Expected slot size for $a_{i}$ when it *changes* its firing phase before *n*-th period (n≥1)
${\tilde{s}}_{nc,i}^{*}\left(n\right)$	Expected slot size for $a_{i}$ when it *does not change* its firing phase until the *n*-th period (n≥1)
${\hat{p}}_{c,j}\left(n\right)$	Probability estimated by $a_{i}$ that a node $c_{i,j}$, caused the collision of $a_{i}$’s firing message in the *n*-th period, *will change* its firing phase in the *n*-th period, where $j=1,\cdots ,{\hat{C}}_{i}\left(n\right)$ (n≥0)
$s_{i}\left(n\right)$	Amount of slot size available to $a_{i}$ up to the *n*-th period(n≥0)
$s_{i,col}\left(n\right)$	Amount of slot size that $a_{i}$ *fails* to transmit data due to the collision up to the *n*-th period (n≥0)
$s_{i,suc}\left(n\right)$	Amount of slot size that $a_{i}$ *succeeds* to transmit data up to the *n*-th period, $s_{i,suc}\left(n\right)=s_{i}\left(n\right)-s_{i,col}\left(n\right)$ (n≥0)

**Table 3:** Comparisons of Desync-TDMA and Ext-Desync-based Proposals.

Features	Proposals																					
	Degesys et al. [10,11]	Hinterhofer et al. [12]	Choochaisri et al. [13]	Lien et al. [14]	Taniguchiet al. [15]	Gao et al. [16]	Ceriotti et al. [17]	Kim, Shin et al. [18]	Yu, Choi et al. [19]	Hyun et al. [20]	Alshudukhi et al. [21]	Ron et al. [22]	Muhlberger et al. [25]	Pagliari et al. [26]	Zheng et al. [27]	Ferrari et al. [28]	Kim, Choi et al. [29]	Yu, Jung et al. [30]	Gentz et al. [31]	Jung et al. [34]	Lee et al. [35]	This work
MH support	X	X	X	X	X	X	X	X	X	X	X	X	O	O	O	O	O	O	O	O	O	O
HTP resolution in MH comm.	X	X	X	X	X	X	X	X	X	X	X	X	O	X	O	X	O	O	O	O	O	O
nodes’ mobility support	X	X	X	X	X	X	X	X	X	X	X	X	X	X	X	X	X	X	X	X	O	O
robustness on firing message collision	X	X	X	X	X	X	X	X	X	X	X	X	X	X	X	X	X	X	X	X	O	O
consideration of PL degradation in resolving collisions	X	O	X	X	X	O	X	X	X	X	X	X	X	X	X	X	X	X	X	X	X	O
consideration of SU degradation in resolving collisions	X	X	X	X	X	X	X	X	X	X	X	X	X	X	X	X	X	X	X	X	X	O
requirement on global time synchronization	X	X	X	X	X	X	X	X	X	X	X	O	X	X	O	X	O	O	X	O	X	X

Note: MH (Multi-hop), HTP (Hidden Terminal Problem), PL (Packet Loss), and SU (Slot Utilization).

## Data Availability

Not applicable.

## Glossary

**Abbreviation**	**Meaning**
BS	Base Station
CSMA/CA	Carrier Sense Multiple Access with Congestion Avoidance
Desync-TDMA	Desynchronization-based TDMA
E2ED	End-to-End Delay
Ext-Desync	Extended Desync-TDMA
MAC	Medium Access Control
MANET	Mobile Ad-hoc Network
MH-Desync	Multi-hop Desync-TDMA
NOMA	Non-Orthogonal Multiple Access
PDR	Packet Delivery Ratio
QoS	Quality of Service
RTS/CTS	Request To Send/Clear To Send
TDMA	Time Division Multiple Access
WSN	Wireless Sensor Network
